# Physiological and mental health changes in cancer patients during the COVID-19 state of emergency

**DOI:** 10.1007/s11332-022-01008-w

**Published:** 2022-10-01

**Authors:** Borja Gutiérrez-Santamaría, Arkaitz Castañeda-Babarro, Maria Soledad Arietaleanizbeaskoa, Nere Mendizabal-Gallastegui, Gonzalo Grandes, Aitor Coca

**Affiliations:** 1 Department of Physical Activity and Sport Sciences, Faculty of Education and Sport, University of Deusto, 48007 Bizkaia, Spain; 2 Primary Care Research Unit of Bizkaia, Biocruces Bizkaia Health Research Institute, Plaza de Cruces 12, 48903 Barakaldo, Biscay Spain; 3 Department of Physical Activity and Sports Sciences, Faculty of Health Sciences, Euneiz University, La Biosfera Ibilbidea, 6, 01013 Vitoria-Gasteiz, Spain

**Keywords:** QLQ-C-30, SF-36, IPAQ-S, Cancer, Physical activity

## Abstract

**Backgrounds:**

Due to the COVID-19 pandemic that we are currently facing, many governments across the world have declared a state of emergency and even confinements. This stressful situation, in addition to prolonged stays at home, may imply a radical change in lifestyle behavior and physical activity (PA). The aim of this study is to evaluate the physiological and psychological effects in cancer patients who changed their PA habits during the COVID-19 state of emergency in Spain.

**Methods:**

Thirty-three participants were evaluated pre- and post-state of emergency. A series of questionnaires was used to assess cancer-specific quality of life.

**Results:**

The most relevant results revealed significantly lower walking time (*p* < 0.001) and sitting time (*p* = 0.014). Upper and lower body strength also decreased significantly (*p* = 0.009 and 0.012, respectively) and oxygen consumption (VO2 peak) (*p* = 0.023). None of the parameters analysed showed significant differences for psychological aspects (QLQ-C-30 and SF-36) and body composition.

**Conclusion:**

Lower physical activity leads to negative physiological adaptation, particularly affecting cardiovascular and strength levels. While it is important to maintain the general population’s amount and intensity of exercise, this particularly vulnerable group’s physical capacity is vital to their health and well-being.

## Introduction

Cancer is one of the principal causes of morbi-mortality in the world with around 19 million new cases recorded in 2020 and population estimates indicate that the number of new cases is expected to rise in the next 2 decades to 29.5 million by 2040 [1]. Almost half of the world population will be diagnosed with cancer at some point in their lives and many of these new cases will receive intensive treatment that will reduce their quality of life and produce functional alterations in other organs, increasing the risk of suffering from other types of diseases [2]. Nevertheless, due to advances in medicine in general, and cancer treatments in particular, there are an increasing number of cancer survivors (CS) (person who suffers from cancer, measured from the moment of the diagnosis until the end of his or her life) [3].

It has been observed that physical inactivity ranks fourth among the risk factors for global mortality (6% of deaths registered worldwide). Additionally, physical inactivity is estimated to be the primary cause of approximately 21 to 25% of colon and breast cancers, 27% of diabetes cases, and approximately 30% of the burden of ischemic heart disease (WHO, 2010).

Exercise contributes to improved health and functional outcomes in the cancer population [4] and, based on review of published evidence regarding the safety and efficacy of exercise in cancer survivors, Schmitz and colleagues state that physical activity (PA) is completely safe and recommend it [2].

A large body of evidence has recommended that cancer patients meet the public health guidelines for PA and the necessary exercise prescription particularly requires consideration of many aspects to positively and safely impact individuals with a cancer diagnosis [5]. Despite being PA recommendations for cancer survivors, they are the same as those set for the healthy population. However, in terms of PA, the same intensity cannot be recommended [6]. In some advanced stages of the disease, cachexia, among other secondary effects of cancer and its treatment, is one of the metabolic multifactorial syndromes that affect a large number of patients. It is caused by a combination of reduced food intake and abnormal metabolism which results in a negative balance of energy and protein synthesis [7].

From the psychological approach, an important revision from the American College of Sport Medicine [2] supports that PA is a consolidated therapy for prevention and management not only in depression and anxiety scenarios but also in several other psychological outcomes like self-esteem and mood during treatment. Moreover, physical exercise program intervention results in statistically significant improvements in quality-of-life (QoL) test scores [8].

Due to the COVID-19 pandemic that we are currently experiencing [9], many governments across the world have declared a state of emergency and even confinements, which is the case of the Government of Spain [10]. This stressful situation, in addition to prolonged stays at home, may imply a radical change in lifestyle behavior and physical activity [11]. However, its impact on the health and well-being of the general population, and the population with cancer in particular, is not known.

The aim of this study is to evaluate the physiological and psychological effect in cancer patients who changed their PA habits during the COVID-19 state of emergency in Spain.

We hypothesise that physiological and psychological parameters worsened during the COVID-19 state of alarm.

## Materials and methods

### Participants

Thirty-three participants, whose characteristics are shown in Table 1, were referred by their oncologists or hematologists at the Cruces, Basurto, and Galdakao hospitals in Bizkaia/Biscay, Basque Country, Spain, as part of the main project called Bizi Orain, which consist in 3 months of physical exercise (progressive resistance and aerobic training) supervised and controlled by professional physicians. Furthermore, the program had different physical and psychological valuations at the moment of the starting the program, at the 3, 6, and 12 months [12].

**Table 1:** Characteristics of the sample

	*N* (%)	Mean±SD	Min-Max
Age (years)	33	55.9±10.9	38–80
Height (cm)	33	162.11±8.30	143–180
Weight (kg)	33	70.50±15.54	51.9–115.1
BMI (kg/m2)	33	26.80±5.43	19.89–43.64
Surgery	25 (75.76)		
Metastatic	8 (24.2)		
Diagnostic			
Breast	18 (54,5)		
Lymphoma	6 (18,2)		
Digestive	4 (12,1)		
Others	5 (15,2)		
Treatment			
Chemotherapy	16 (48,5)		
Radiotherapy	2 (6,1)		
Hormone therapy	1 (3)		
Chemotherapy+radiotherapy	8 (24,2)		
Chemotherapy + radiotherapy + hormone therapy	2 (6,1)		
Radiotherapy+ Hormone therapy	4 (12,1)		
Sex			
Female	28 (84.8)		
Male	5 (15.2)		

Notes: *SD* standard deviation; *Min* minimum; *Max* Maximum; *N* number of participants; *BMI* Body mass index

All patients were participants in a physical exercise intervention program Bizi Orain [12] for cancer patients. Although they had completed the first physical evaluation, they did not begin the training program due to the COVID-19 state of emergency declared in Spain. This evaluation consisted of pre-state of emergency measures of the different parameters in our study. After the state of emergency was lifted and once safety was guaranteed, the assessments were performed again to begin with the program. Thus, these are the assessments used as post-state of emergency for the present study.

Inclusion and exclusion criteria followed our protocol [12]. All the subjects were natives of Spain. The study protocol was conducted ethically according to international as well as journal standards [13] and was approved by the Institutional Research Ethics Committee (PI2019016). The trial was registered on January 18, 2019 (Clinical Trials.gov NCT03819595).

### Measurement: outcome variables

Patients were physically and psychologically evaluated at the University of Deusto, (Biscay, Spain) at the same times of day (9:00 to 14:00) and in similar environmental conditions (temperature, ± 21 °C; relative humidity, 50–55%; barometric pressure, ± 720 mmHg).

**Fig. 1:**
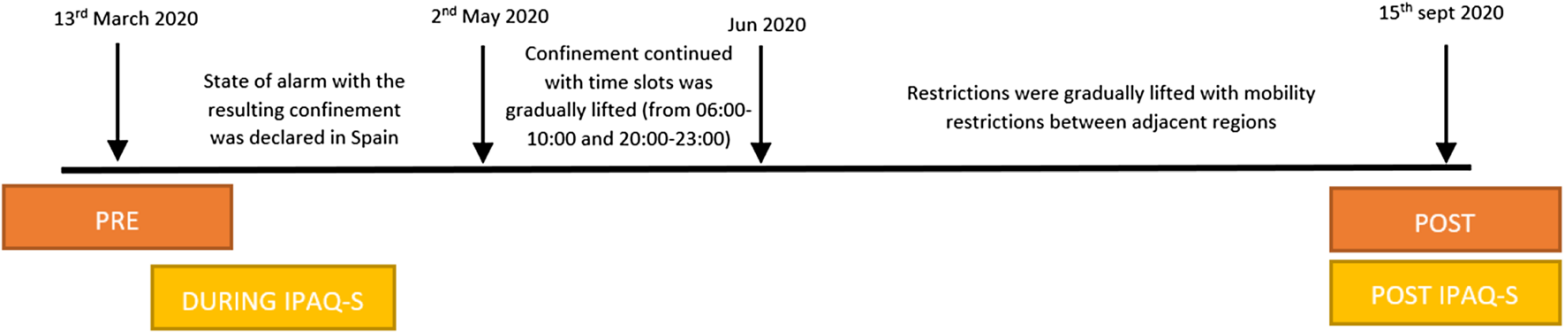
Evolution of phases during the COVID-19 pandemic and restrictions

On 14 March 2020, a state of alarm with the resulting confinement was declared in Spain and remained in effect until 21 June 2020, when restrictions were gradually lifted, but maintaining mobility restrictions, closure of sports facilities, gymnasiums, and nightlife venues. Curfews were imposed from 22:00 to 6:00 [10]. These restrictions may have influenced the amount of physical activity (Fig. 1).

The questionnaire used to evaluate the levels of PA was the IPAQ short version (IPAQ-S) validated in Spanish (Wolin et al. 2008) which asks about three specific types of activity undertaken during the previous 7 days in the four domains (leisure time, work, household activities, and transport). An IPAQ-S during and post-state of emergency activity was completed when the evaluation was performed after the COVID-19 restrictions.

The subjects’ height was measured using a wall stadiometer (Seca, Germany) and body composition with an Inbody 770 bioimpedance analyzer (In-body, Seoul, Korea). Resting heart rate and blood pressure Omron X3 Comfort (HEM-7155-EO) (OMRON, Kyoto, Japan) were measured seated in a quiet room.

To determine the VO2peak (the peak VO2 value in the last 30 s of the last stage of the test sub-maximal test perform by the subjects), a test performed on an electric braking cycle-ergometer (Ergostik, Geratherm Respiratory, Bad Kissingen, Germany). Following an unloaded 5-min warm-up, the load was increased 10 W per minute starting from an initial load of 20 W. Participants were instructed to maintain cadence over 65 rpm. Gas exchange was analysed throughout the test with a gas analyser (Ergostik, Sanro, Spain). The first and second ventilatory thresholds (VT1 and VT2) were obtained using the first exponential increase in the oxygen (O2) ventilatory (VE) equivalent (VE/VO2). The VT2 or respiratory compensation point (RCP) was determined using the ventilatory equivalent method (VE/VCO2) ratio [6, 14]. The test was carried out until the subjects reached their VT2 or heart rate at 85% of their theoretical maximum.

Through the test of five maximum repetitions (5RM) and using strength exercise machines, general muscular strength was evaluated with chest press exercises (L070, BH, Spain) and leg press (L050, BH, Spain). The protocol used was the one previously applied [15], consisting of 10 repetitions warm-up with an easy weight (~ 50% of 5RM) and the 3–5 progressive 5RM until exhaustion (~ 65,75,85,95% of 5RM).

A series of questionnaires was used to assess cancer-specific quality of life. Cancer-specific quality of life is evaluated by the European Organization for Research and Treatment of Cancer (EORTC QLQ-C-30) questionnaire [16] scaled from 1 to 100, and higher scores represent greater function/quality of life. This questionnaire includes five functional domains (physical, cognitive, emotional, and social role; higher scores represent greater function/quality of life) and three symptom scales (fatigue, pain, and nausea; lower scores indicating a higher quality of life/less symptom severity). The Medical Outcomes Study 36-Item Short-Form Health Survey (SF-36) (scaled from 1 to 100, higher scores indicating greater quality of life) was used to assess general health-related quality-of-life status across physical functioning, physical role functioning, bodily pain, general health, vitality, social functioning, emotional role functioning, and mental health domains [17].

## Statistical analysis

T test was used to examine differences in physiological parameters (musculoskeletal mass, fat mass, visceral fat, Wmax, WVT2, and Upper and Lower body strength) and psychological parameters of quality of life (EORTC-QLQ-30 and SF-36) in two time periods (time: pre- versus post-pandemic restrictions). For the psychometric analyses, grouping and scaling based on the original papers [18, 19] were assessed for the multi dimensions of the questionnaires and Cronbach´s Alpha reliability test was used prior to the analysis. All analyses were performed in SPSS v.26 with alpha level set at 0.05.

## Results

As shown in Table 2, body composition values remained similar both before and after the state of alarm. Musculoskeletal mass measures did not evidence significant differences (*p* = 0.934). The percentage of fat mass presented a slight insignificant decrease (*p* = 0.354), and finally, visceral fat also showed an insignificant reduction (*p* = 0.791).

**Table 2:** Body composition levels and physical and physiological parameters pre and post the break in PA due to the state of emergency

Variable	Pre (Mean ± SD)	Post (Mean ± SD)	*P* value	*d*-Cohen
Musculoskeletal mass (kg)	23.8 ± 5.4	23.8 ± 5.6	0.934	0
Fat mass (%)	37.1 ± 8.4	35.5 ± 10.7	0.354	0.16
Visceral fat (cm2)	134.2 ± 54.2	132.4 ± 55.3	0.791	0.03
VO2peak (mL∙min∙kg^ − 1^)	17.03 ± 5.08	15.42 ± 3.98	0.023*	0.35
WMAX (W)	90.34 ± 28.85	86.55 ± 27.03	0.078	0.14
WVT2 (W)	88.57 ± 26.06	85 ± 26.60	0.106	0.13
Lower body strength (kg)	61.96 ± 22.10	52.86 ± 23.18	0.012*	0.40
Upper body strength (kg)	30.39 ± 14.46	24.95 ± 14.27	0.009*	0.38

*p < 0.05; *WMAX* Watts maximum; *WVT2* Watts at ventilatory threshold 2

Table 3 shows the differences in PA, sitting time (ST), and walking time (WT). Regarding vigorous physical activity (VPA) and moderate physical activity (MPA), no significant differences were observed between the activity measurements during and after the state of emergency. It should be noted that no patient except one reported vigorous-intensity activity during the state of emergency and no patients reported VPA post-state of emergency. Alternatively, with walking and sitting time, significant differences were reported in both variables with *p* values < 0.001 and 0.014, respectively. Meanwhile, considerable growth was observed in the number of hours invested in walking in comparison to such time during the state of emergency (*d* = 1.51). As for the sitting hours, a slight but significant decrease was observed after the state of emergency.

**Table 3:** Variations in levels of PA and time sitting during and after the state of emergency due to COVID-19.

Variable	During	Post	*P* value	*d*-Cohen
VPA (min/week)	1.88 ± 10.61	0	0.325	< 0.01
MPA (min/week)	117.50 ± 160.02	91.56 ± 124.24	0.349	0.18
Walking time (min/week)	76 ± 113.08	327.50 ± 206.92	< 0.001*	1.51
Sitting time (hours/day)	7.53 ± 2.66	6.63 ± 5	0.014*	0.47

*VPA* vigorous physical activity; *MPV* moderate physical activity

(*p < 0.05)

Large but insignificant decreases (*p* = 0.0789) were found for cardiovascular condition (Table 2) in the amount of Wmax the patients were capable of moving. In turn, the measure of the VT2 watts was also slightly lower after the state of emergency. However, in spite of observing a slight watt decrease of 4.1%, the reduction (*p* = 0.106) is not statistically significant. The last physiological parameter for cardiovascular function measured by peak oxygen consumption (VO2peak) shows a significant decrease (*p* = 0.023), evidenced by *n* = 20 who reduced their VO2peak and n = 8 who raised it. Finally, upper and lower body strength levels were significantly lower (*p* = 0.012 and *p* = 0.009, respectively).

Finally, the results shown in Table 4, obtained with the SF-36 and EORTC-QLQ-30 questionnaires, did not evidence significant differences in any of the variables measured therein.

**Table 4:** Psychological differences between pre- and post-state of emergency due to COVID and their significance (*p* value)

Variable	Pre	Post	*P* value	*d*-Cohen
EORTC-QLQ-30				
Global health status	4.74 ± 1.15	4.83 ± 1.19	0.684	0.08
Emotion role	1.56 ± 0.63	1.55 ± 0.56	0.798	0.02
Social function	1.56 ± 0.83	1.67 ± 0.92	0.408	0.12
Cognitive function	1.53 ± 0.71	1.54 ± 0.75	0.851	0.01
SF-36				
Emotional role	1.77 ± 0.39	1.87 ± 0.38	0.096	0.26
Mental health	4.26 ± 0.82	4.14 ± 0.75	0.568	0.15

EORTC QLQ-C-30: European Organization for Research and Treatment of Cancer; SF-36: Short-Form Health Survey

## Discussion

Fulfilling the objective of the research to observe the changes produced in terms of PA during the time of the state of alarm by COVID-19 and its implication in physiological and psychological aspects, the most relevant results revealed significantly lower WT and ST. Upper and lower body strength also decreased significantly. None of the parameters analysed showed significant differences for psychological aspects.

No modifications in body composition were found between the pre- and post-state of emergency levels. The patients’ body composition may already have been modified by their disease, in which higher fat mass and lower muscle mass are often observed [20].

There is a growing body of evidence noting less physical activity during a state of alarm with restricted mobility such as that caused by COVID-19. For this reason, there are now systematic reviews on the general population’s level of physical activity during the recent state of emergency [21]. The literature indicates less physical activity and more sitting time.

The sample studied did not show significant differences in VPA, which may be explained by the low levels of intense activity due to the pathology itself which induces chronic fatigue that could hinder exercise [22]. Due to the pathology, these patients are sometimes recommended to stop VPA given the risk of carrying out these activities without professional supervision, and that is why, many doctors advise against carrying out this type of intense practice [23]. However, breaking out of this vicious cycle and beginning to exercise are a good strategy to fight cancer-related chronic fatigue [24]. It is important to note that our sample does not reach the 75 min/week minimum levels of vigorous activity recommended by the WHO. While these are recommendations for the healthy population, compliance with the VPA and MPA guidelines tends to improve the prognosis of persons with pathologies, and particularly cancer survivors [25], 26. Intense or high-intensity activities are advisable as they result in adaptation similar to that generated by longer periods of moderate exercise but require less time (40% of the time). We could therefore state that vigorous exercise is more effective for achieving beneficial physicological adaptation, and is a safe option [25, 27, 28].

Higher levels have been noted with MPA and this indicates that this group does more MPA than VPA, although they do not reach the WHO’s recommended minimum guidelines of > 150 min/week MPA [29]. The time devoted to walking was reduced during the state of emergency, possibly due to confinement (during the state of alarm) or restricted mobility between areas. However, once the mobility restrictions were lifted, a sharp rise in walking time was noted. The logical consequence of restrictions on mobility during the state of alarm would be for walking time to decrease, and likewise for walking time to increase as restrictions were lifted.

This may have been due to the subjects’ perception that being outdoors involved a lower risk of COVID-19 infection. In addition, another important factor is the recommendation that the doctor gives to these patients to walk [30], age is also a factor that makes patients walk more and perform less VPA or MPA [31].

As walking time was reduced, sitting time increased and then fell once again when walking time increased.

Although our sample is formed by a population that is being or has been treated for cancer, some similar patterns have been observed in a study with 3800 healthy subjects before and after the state of emergency. Performed by Spanish researchers, it showed lower VPA, MPA, and walking time but higher sitting time [11]. This behavior related to time devoted to physical activity was confirmed through reviews of articles on the effects of confinement on physical activity [21].

Lower physical activity leads to negative physiological adaptation, particularly affecting cardiovascular and strength levels. According to their age and prognosis, patients may even lose their autonomy [32].

Oxygen consumption is a predictor of survival in the general population [33, 34] and in the cancer population [2, 35]. We found a significantly lower V02peak during the period studied. The V02peak depends on three main components: oxygen uptake, the blood’s oxygen-carrying capacity to the muscles, and mitochondrial functionality [36]. When observing the effects of lack of exercise on the V02peak components, we find that 2 days of no exercise lowers plasma levels by 5 to 12% [37]. An 8% drop in cardiac output, responsible for carrying oxygen, was noted, together with other cardiac modifications after 21 days with no exercise training [38]. Muscular capillarisation also fell to pre-exercise training levels in just 4 weeks [39]. Finally, a 12 to 28% drop in mitochondrial production of ATP was noted 3 weeks after stopping exercise [39]. 8 weeks of combined exercise training followed by 8 weeks of no exercise in breast cancer patients evidenced 8% lower VO2peak for cardiovascular levels [40]. Seeing that the variations are important in a relatively short period of time in this patient is important to not been more than few days without exercise to maintain the adaptations. In turn, decreased PA led to significantly lower strength levels during the COVID-19 state of alarm.

The articles by Mujika and Padilla [41, 42] explain how strength levels can drop by 7–12% during an 8 to 12 week period of no exercise training. Although these patients were not totally inactive, their strength levels fell (14.68% for lower body and 17.9% for upper body) more than expected for the exercise population in the same situation. Mujika and Padilla's article is carried out in a young athlete population, and seeing that it does not differ much from our subjects, we could identify a pattern of loss of strength regardless of the age and sex of the subjects. It is true that the older you are, the loss of muscle mass and strength would be increased, but in this case, it behaves in a similar way. This loss may have been due to their treatment, although muscle mass loss was not found as was anticipated for these patients and their treatment [43, 44]. In line with this evidence, our study sample’s strength levels decreased around 14 to 15%, which is similar to the losses expected in the healthy exercise population. The study cited above [40] found lower strength levels similar to the findings in our sample of cancer patients. Such reduced strength levels might have been due to neuronal disadaptation caused by lack of strength training as the peripheral adaptations (amount of muscle mass) did not change.

No significant psychological differences were observed in any of the variables measured with the SF-36 and EORTC QLQ-30 questionnaires. This may be explained by the negative results that these patients show on this type of questionnaire measuring quality of life, since their health problems cause them to have a poor perception of this aspect [45]. In studies on cancer patients, physical exercise was found to significantly improve the global health status measured by the EORTC-QLQ-30 questionnaire, although there were no significant differences when pre-intervention values were compared with those recorded during the state of emergency. No significant differences were found in any of the items when comparing pre-exercise training (without having begun the exercise programme) and the findings obtained after a state of emergency. Thus, the conclusion is that subjective values for quality of life would return to their initial levels after a state of emergency [40]. The emotional state item showed a tendency toward significance. The respondents’ subjective assessment of their emotional state could have become worse due to the restrictions imposed during the state of alarm, which is the case of healthy people who evidenced greater anxiety, depression, and emotional impact.

In general, the behavior of the healthy population in the exceptional situation of COVID-19 had been studied, but little was known about cancer patients regardless of sex and age. Many parameters (nutrition, genetics, socio-economic situation, etc.) could have affected the results of our study, but we have been able to show how the results are in line with the results obtained in a healthy population.

## Conclusion

 Aware of the role that PA plays in individuals’ health, functioning as a polypill with broad benefits in the case of chronic diseases [46], it is also important to highlight the importance of PA to the cancer population. While no differences in body composition or psychological state were found in our sample, possibly due to this group’s poor parameters to begin with, there was a decline in strength and fitness level as well as less time of PA levels during this period. Attention should focus on maintaining or increasing PA levels in the event of another state of alarm in the future, because while it is important to maintain the general population’s amount and intensity of exercise, this particularly vulnerable group’s physical capacity is vital to their health and well-being [25] Even if there are no significant changes in psychological aspects, it is an important aspect to consider during treatment to provide them with the necessary help.

## Strengths and limitations

This is one of the first studies that measures changes in lifestyle and psychological variables in cancer patients due to the new situation generated by the COVID-19 pandemic, considering psychological and physiological aspects.

 The main limitations of this study are that the study was a part of larger project that was being conducted as the COVID-19 pandemic started. As such, the sample of this study was the patients that were evaluated to start the research project and did not initiate any activities. These patients were contacted again to start the project once it was allowed and we retested them. This explains that the sample is small and heterogenous (with a large age dispersion that has to be taken care of when reading the results), besides the study was not randomized. We also could not compare with a control group because of the nature of the study, and the COVID-19 was the same for all the patients.
